# Beneficial Effect of Dimethyl Fumarate Drug Repositioning in a Mouse Model of TDP-43-Dependent Frontotemporal Dementia

**DOI:** 10.3390/antiox13091072

**Published:** 2024-09-02

**Authors:** Ignacio Silva-Llanes, Raquel Martín-Baquero, Alicia Berrojo-Armisen, Carmen Rodríguez-Cueto, Javier Fernández-Ruiz, Eva De Lago, Isabel Lastres-Becker

**Affiliations:** 1 Instituto de Investigaciones Biomédicas “Alberto Sols” UAM-CSIC, Arturo Duperier, 4, 28029 Madrid, Spain; ignaciosilva@iib.uam.es (I.S.-L.); alicia.berrojo@salud.madrid.org (A.B.-A.); 2 Instituto de Investigación Sanitaria La Paz (IdiPaz), 28029 Madrid, Spain; 3 Departamento de Bioquímica y Biología Molecular, Instituto Universitario de Investigación en Neuroquímica, Facultad de Medicina, Universidad Complutense, 28040 Madrid, Spain; raqmar11@ucm.es (R.M.-B.); carc@med.ucm.es (C.R.-C.); jjfr@med.ucm.es (J.F.-R.); elagofem@ucm.es (E.D.L.); 4 Instituto Ramón y Cajal de Investigación Sanitaria (IRYCIS), 28040 Madrid, Spain; 5 Centro de Investigación Biomédica en Red de Enfermedades Neurodegenerativas (CIBERNED), Instituto de Salud Carlos III, 28031 Madrid, Spain; 6 Department of Biochemistry, School of Medicine, Universidad Autónoma de Madrid, 28029 Madrid, Spain; 7 Institute Teófilo Hernando for Drug Discovery, Universidad Autónoma de Madrid, 28029 Madrid, Spain

**Keywords:** NRF2, TDP-43, DMF, neurodegeneration, neuroinflammation

## Abstract

Frontotemporal dementia (FTD) causes progressive neurodegeneration in the frontal and temporal lobes, leading to behavioral, cognitive, and language impairments. With no effective treatment available, exploring new therapeutic approaches is critical. Recent research highlights the transcription factor Nuclear Factor erythroid-derived 2-like 2 (NRF2) as vital in limiting neurodegeneration, with its activation shown to mitigate FTD-related processes like inflammation. Dimethyl fumarate (DMF), an NRF2 activator, has demonstrated neuroprotective effects in a TAU-dependent FTD mouse model, reducing neurodegeneration and inflammation. This suggests DMF repositioning potential for FTD treatment. Until now, no trial had been conducted to analyze the effect of DMF on TDP-43-dependent FTD. In this study, we aimed to determine the potential therapeutic efficacy of DMF in a TDP-43-related FTD mouse model that exhibits early cognitive impairment. Mice received oral DMF treatment every other day from presymptomatic to symptomatic stages. By post-natal day (PND) 60, an improvement in cognitive function is already evident, becoming even more pronounced by PND90. This cognitive enhancement correlates with the neuroprotection observed in the dentate gyrus and a reduction in astrogliosis in the stratum lacunosum-moleculare zone. At the prefrontal cortex (PFC) level, a neuroprotective effect of DMF is also observed, accompanied by a reduction in astrogliosis. Collectively, our results suggest a potential therapeutic application of DMF for patients with TDP-43-dependent FTD.

## 1. Introduction

Frontotemporal dementia (FTD) is a progressive neurodegenerative disease affecting 4.1 per 100,000 individuals, typically between the ages of 45 and 65. Clinically, FTD patients exhibit personality, cognitive, behavioral, and language disturbances due to the degeneration of the frontal and temporal lobes, followed by hippocampal atrophy [1]. While Alzheimer’s disease (AD) is the most common form of dementia, FTD is the second leading cause in adults and the most common in those under 65. The etiology of FTD is complex and heterogeneous, with approximately 50–60% of cases being sporadic and the rest having a familial origin, linked to mutations in genes such as MAPT, TARDBP, GRN, C9orf72, and VCP. FTD is characterized by toxic, ubiquitin-positive protein aggregates [2]. Based on the predominant protein, FTD is classified into subtypes, mainly FTD-TAU and FTD-TDP-43, each affecting about 45% of patients.

TDP-43 is encoded by *TARDBP* and is a nuclear RNA/DNA-binding protein essential for RNA metabolism, including stability, transport, transcription, and processing. In FTD, TDP-43 mislocalizes from the nucleus to cytoplasmic inclusions, suggesting that either loss of normal function or gain of toxic properties play a key role in the disease’s pathogenesis. There are currently no approved treatments to slow FTD progression; existing strategies focus on symptom management. However, pharmacologically modulating TAU and TDP-43 proteins could be promising. The transcription factor NRF2 has emerged as crucial in limiting neurodegeneration, affecting processes like proteostasis, oxidative stress, and neuroinflammation. NRF2 levels are typically low due to different regulatory pathways. The primary mechanism regulating NRF2’s transcriptional activity involves its binding to the E3 ligase adapter Kelch-like ECH-associated protein 1 (KEAP1). This interaction presents NRF2 for ubiquitination by Cullin 3 and RING-box protein 1 (CUL3/RBX1), leading to its subsequent degradation by the proteasome [3,4,5]. Under normal homeostatic conditions, NRF2 levels are very low. However, modifications of key cysteine residues in KEAP1 by electrophiles or reactive oxygen species (ROS) cause conformational changes that prevent KEAP1 from targeting NRF2 for degradation. This results in the accumulation of newly synthesized NRF2, which can then translocate to the nucleus and bind to an enhancer sequence, known as the antioxidant response element (ARE), in the promoter regions of NRF2 target genes [6]. Together with members of the small musculo-aponeurotic fibrosarcoma (MAF) family, NRF2 recruits additional components of the transcriptional machinery. Another mechanism for modulating NRF2 involves the E3 ligase adapter β-transducin repeat-containing E3 ubiquitin-protein ligase (β-TrCP), which presents NRF2 to a CUL1/RBX1 complex [7,8]. This leads to an alternative pathway for the ubiquitin-dependent proteasome degradation of NRF2. Thus, NRF2 is subject to multiple levels of regulation, in addition to the numerous pathways modulated downstream of its activation.

Among NRF2 activators, dimethyl fumarate (DMF) has shown neuroprotective effects and is already approved for relapsing-remitting multiple sclerosis as Tecfidera, despite some gastrointestinal side effects. In our research group, we have previously shown that in FTD-TAU models, hippocampal cells overexpressing the human mutant TAU^P301L^ protein produce the chemokine fractalkine CXCL1. This chemokine activates AKT in microglia, inhibits glycogen synthase kinase-3β (GSK-3β), and upregulates the transcription factor NRF2/*NFE2L2* and its target genes, including heme oxygenase 1 (*Hmox1*) [9]. Additionally, DMF treatment mitigated neurodegenerative features in our FTD-TAU mouse model, reducing TAU hyperphosphorylation, neurodegeneration, neuroinflammation, and oxidative stress, supporting the repurposing of DMF for FTD treatment [10]. These results clearly demonstrated the importance of pharmacological modulation of NRF2 in FTD-TAU. However, regarding TDP-43 protein associated with FTD, evidence for NRF2 transcription factor involvement in this pathology is still limited. Recently, our group reported that in a transgenic FTD model with TDP-43 protein overexpression in forebrain neurons (FTD-TDP-43), there was only a significantly decreased expression of the NRF2-dependent enzyme NQO1 in the prefrontal cortex (PFC) of these mice, which cannot be attributed to alterations in the NRF2 pathway [11]. However, in these mice, we cannot rule out a neuroprotective effect of the NRF2 inducer, DMF, on the neurodegeneration and neuroinflammation observed in mice overexpressing TDP-43. Therefore, this study focuses on analyzing the potential beneficial effects of DMF treatment in the prefrontal cortex and hippocampus of FTD-TDP-43 mice.

## 2. Materials and Methods

### 2.1. FTD Mouse Model: CaMKII-TDP-43

All experiments were conducted with male animals that had PND90 as the endpoint age, with FVB-N background FVB-N/CaMKII-TDP43 transgenic and non-transgenic mice bred in our animal facilities from initial breeders provided by Dr. Che-Kun J Sheen (The PhD Program for Neural Regenerative Medicine, Taipei Medical University, Taiwan) and previously characterized [12]. This model is based on the overexpression of the TDP-43 protein under the control of the Ca^2+^/calmodulin-dependent protein kinase II (CaMKII) promoter to specifically and selectively overexpress TDP-43 in forebrain neurons. CaMKII-TDP-43 male mice and their wildtype animals were generated from homozigotic breeding. Animals were housed in the animal facilities of Complutense University (CAI-Animalario, Faculty of Medicine, Complutense University, ref. ES280790000086) under controlled photoperiod (08:00–20:00 light) and temperature (22 ± 1 °C) and with free access to a standard diet and water. DMF (100 mg/kg) (Merck Sigma-Aldrich, Buchs, Switzerland) was suspended in 0.8% methocel (Sigma-Aldrich) and given by oral gavage. This dose was established based on previous results from our group, where neuroprotective effects were observed in other neurodegenerative disease mouse models [10,13]. We did not detect significant weight loss, hair loss, or other gross alterations in the DMF-treated mice during the administration period.

### 2.2. Behavioral Analysis

To evaluate recognition memory, the novel object recognition (NOR) test was performed as described [14]. Briefly, on the first and second day, mice were allowed to explore an empty open field (an opaque methacrylate box (50 × 50 × 50 cm) with a base covered with sawdust) for 10 min to habituate to the field. On the third day, mice were placed in the open field with two identical objects to explore for 10 min. On the fourth day, mice were placed in the open field with one of the objects replaced by a new object and explored for 10 min. Time spent exploring either the novel object (TN) or the familiar object (TF) was recorded. Differences were represented as a discrimination index (DI = (TN − TF)/(TN + TF)). The test was performed at two different times: first, at an early symptomatic stage (PND60), and second, 24 h before sacrifice (PND90), with 11–12 animals from each experimental group. 

### 2.3. Randomization and Blinding

Animals were randomized for treatment. Data collection and evaluation of all experiments were performed blindly of the group identity. The data and statistical analysis are presented in line with the recommendations on experimental design and analysis in pharmacology [15].

### 2.4. Analysis of mRNA Levels via QUANTITATIVE Real-Time PCR

Total RNA extraction, reverse transcription, and quantitative polymerase chain reaction (qPCR) were performed as detailed in previous articles [11] Primer sequences are shown in Table 1. Data were analyzed using the 2^−ΔΔCT^ method, with normalization of the raw data based on the geometric mean of *Tbp*, *Actb*, and *Gapdh* (Merck Sigma-Aldrich, Darmstadt, Germany), encoding housekeeping proteins. All PCR amplifications were performed in triplicate.

### 2.5. Immunofluorescence on Mouse Tissues

Immunofluorescence assays were performed on 30-µm thick coronal brain sections at the hippocampus and prefrontal cortex (PFC). The protocol followed was previously described [16]. Primary and secondary antibodies are described in Table 2. The area of the dentate gyrus from mice treated with vehicle or DMF stained with DAPI or anti-CALBINDIN D28K was analyzed by the ImageJ program. A total of three images per side and condition were analyzed as follows. The images were transformed into 16-bit with the ImageJ program. Then, with the “Free Hand Selection” tool of the ImagenJ program, we manually selected only the dentate gyrus area of each image stained with DAPI or anti-CALBINDIN D28K. The dimension of the dentate gyrus inside the selected area was quantified using the “Measure” tool in the ImageJ program and the raw results, measured in inches, were represented. 

### 2.6. Stereological Analysis of Microgliosis and Astrogliosis

Cell counts were performed every eight sections (30 µm-thick) using Fiji Software v1.54f in three sections of the hippocampus or PFC. The error coefficient attributable to the sampling was calculated according to Gundersen and Jensen (1987) [17], and values ≤0.10 were accepted. (n = 4–5 animals per experimental group).

### 2.7. Statistical Analyses

Data are presented as the mean ± SEM. To determine the statistical test to be used, we employed GraphPad Instat 3, which includes the analysis of the data to a normal distribution via the Kolmogorov–Smirnov test. In addition, statistical assessments of differences between groups were analyzed (GraphPad Prism 8 by Dotmatics, San Diego, CA, USA) by performing an unpaired Student’s *t-*tests. A one-way ANOVA with post-hoc Newman–Keuls test was used.

## 3. Results

In the FTD CaMKII-TDP-43 mouse model, overexpression of the TDP-43 protein in neurons of the prefrontal cortex (PFC) and hippocampus has been shown to replicate disease-specific changes, including cognitive impairments, pathological mislocalization of TDP-43, and increased gliosis [12,14,18]. Therefore, we aim to investigate whether repositioning DMF treatment in this transgenic model for TDP-43-dependent FTD could modulate the disease’s degenerative progression.

### 3.1. DMF Treatment Alleviates Cognitive Impairment Caused by TDP-43

**Figure 1:**
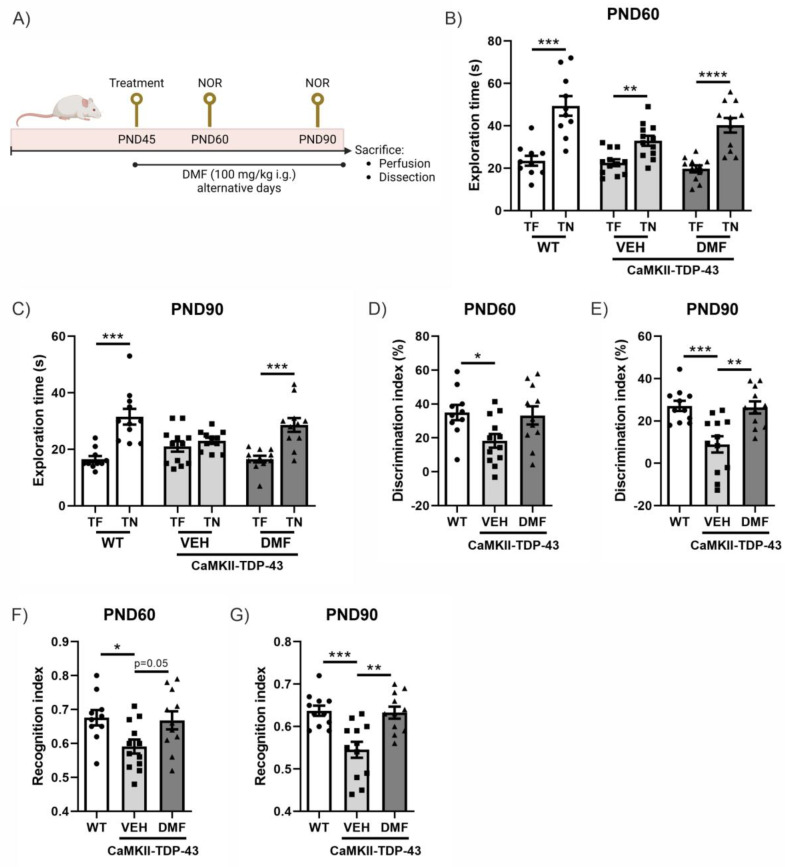
Response in the Novel Object Recognition (NOR) test of CAMKII-TDP-43 and WT mice at PND60 and PND90 after a chronic i.g. administration of DMF (100 mg/kg) from PND45 up to PND90 (**A**) Timeline representation of the experimental design: At PND45, we started the treatments (VEH, or DMF 100 mg/kg, i.g., respectively). At PND60, we performed the first NOR analysis (four consecutive days), and at PND90, before sacrifice, we performed another NOR test. (**B**,**C**) Analysis of the exploration time of familiar object (TF) and novel object (TN) at PND60 and PND90. (**D**,**E**) Analysis of the discrimination index at PND60 and PND90. (**F**,**G**) Analysis of the recognition index at PND60 and PND90. Bars indicate the mean of n = 10–12 samples ± SEM. Asterisks show significant differences with * *p* < 0.05, ** *p* < 0.01, *** *p* < 0.005, **** *p* < 0.001 comparing each group according to a one-way ANOVA followed by Tukey’s post-test.

In this murine model of FTD associated with TDP-43, it has been previously described that cognitive impairment already occurs at PND60 and is exacerbated at PND90 [14,18]. Therefore, we analyzed the effect of DMF treatment at these two ages (Figure 1A). CaMKII-TDP-43 animals showed lower exploration times compared to the WT animals at both ages (Figure 1B,C). This resulted in an important decrease in the discrimination index (Figure 1D,E) and preference index (Figure 1F,G) in CaMKII-TDP-43-VEH mice compared to WT animals. Treatment with DMF produced a significant improvement in CaMKII-TDP-43-DMF animals, both in terms of the discrimination index (Figure 1D,E) and the preference index (Figure 1F,G), highlighting the treatment’s potential to ameliorate cognitive deficits in this animal model.

### 3.2. Neuroprotective Effect of DMF on the Granular Layer of the Hippocampus

We examined whether DMF treatment had crossed the blood-brain barrier and activated the transcription factor NRF2 pathway in the hippocampus (Figure S1) of CaMKII-TDP-43 mice by determining the mRNA levels of various NRF2-dependent enzymes. Since the mice were sacrificed 24 h after the last treatment dose, we observed only a modest induction of late-kinetic NRF2-dependent enzymes, such as *Nqo1* (Figure S1C) and *Gpx1* (Figure S1D), compared to the vehicle-treated CaMKII-TDP-43 mice. We confirmed that TDP-43 overexpression decreases mRNA levels of *Nqo1* in transgenic mice, and that DMF treatment normalizes and even increases *Nqo1* expression to baseline levels.

**Figure 2:**
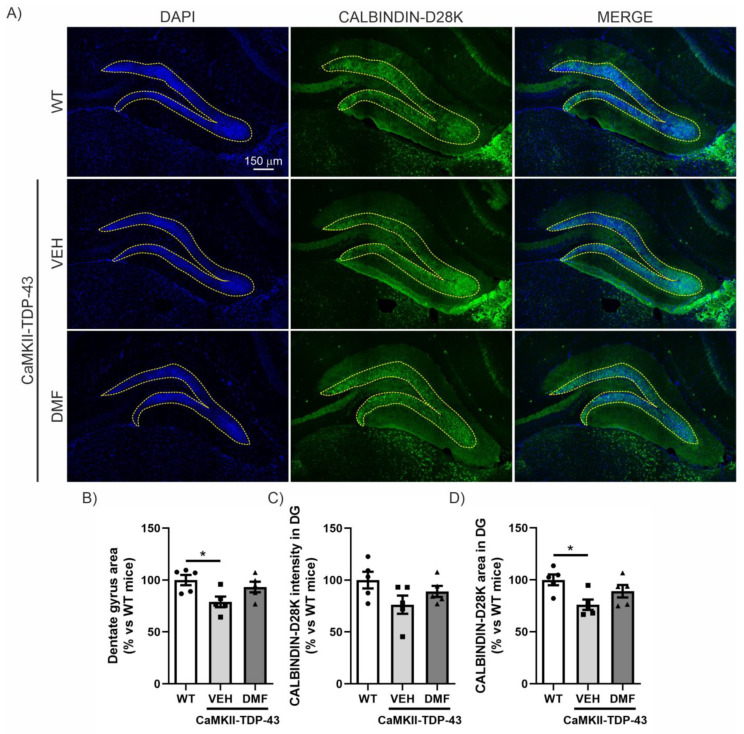
DMF has a neuroprotective effect on the granular cell layer of the dentate gyrus. (**A**) Immunofluorescence staining of DAPI and CALBINDIN-D28K of 30 μm-thick sections of the dentate gyrus of the hippocampus from WT, and CAMKII-TDP-43 mice treated with vehicle or DMF. (**B**) Quantification of the area stained with DAPI in the dentate gyrus from WT, and CAMKII-TDP-43 mice treated with vehicle or DMF. (**C**) Quantification of the intensity of the area stained with CALBINDIN-D28K in the dentate gyrus from WT, and CAMKII-TDP-43 mice treated with vehicle or DMF. (**D**) Quantification of the area stained with CALBINDIN-D28K in the dentate gyrus from WT, and CAMKII-TDP-43 mice treated with vehicle or DMF. Bars indicate the mean of n = 4–5 samples ± SEM. Asterisks show significant differences with * *p* < 0.05 comparing each group according to a one-way ANOVA followed by Tukey’s post-test.

Within the hippocampus, the dentate gyrus (DG) is believed to contribute to the formation of new episodic memories [19], spontaneous exploration of novel environments, and other functions. Therefore, we investigated whether there was a correlation between the data obtained from the NOR (Figure 1) and structural alterations in the DG. DAPI staining, which binds strongly to adenine-thymine rich regions in DNA, indicated that TDP-43 overexpression induced partial loss of the granular cell layer in CaMKII-TDP-43 mice (Figure 2A,B). However, this loss was partially attenuated by the treatment with DMF, as shown in the area quantification (Figure 2B). Regarding synaptic plasticity, we evaluated the expression levels of CALBINDIN-D28K, a member of the calcium-binding protein superfamily, that, in the hippocampus, has been found in glutamatergic neurons, including mature granule cells in the DG [20]. This protein localizes to axonal boutons and dendritic spines and dynamically modulates synaptic plasticity; reduced CALBINDIN-D28K levels correlate with impaired hippocampus-dependent memory [21]. Immunofluorescence analysis of the DG showed reduced CALBINDIN-D28K expression levels (Figure 2A–C) and area (Figure 2A–D) in the granular layer of CaMKII-TDP-43 mice (Figure 2A–C) compared to WT animals, and the treatment with DMF was able to partially recover it. These results suggest that treatment with DMF provides protection against TDP-43-induced neurodegeneration.

Regarding other areas of the hippocampus, such as CA1, CA2, and CA3, we also conducted an analysis of the neurodegeneration process by counting the number of neurons and by assessing CALBINDIN-D28K levels. In the CA1 area, we observed that CaMKII-TDP-43 mice have a slight neuronal loss compared to WT mice, and DMF treatment only has a subtle effect (Figure S2A–C). In the CA2 area, we did not observe any neuronal damage effects due to TDP-43 overexpression (Figure S2D–F). Finally, in the CA3 area, there is a slight decrease in both the number of neurons and CALBINDIN-D28K levels in CaMKII-TDP-43 mice, and DMF treatment does not appear to have any effect (Figure S3). Taken together, these results suggest that the CaMKII-TDP-43 mice exhibit cognitive impairment associated with degeneration of the DG, and that DMF treatment can mitigate this degenerative condition, having a positive impact on their cognition.

### 3.3. DMF Treatment Partially Reverses Astrogliosis but Not Microgliosis in the Hippocampus of CaMKII-TDP-43 Mice

One of the main characteristics of FTD-TDP-43 is neuroinflammation, which involves reactive astrogliosis and microgliosis accompanied by an imbalance between pro-inflammatory cytokines and anti-inflammatory (homeostatic) factors [22,23].

**Figure 3:**
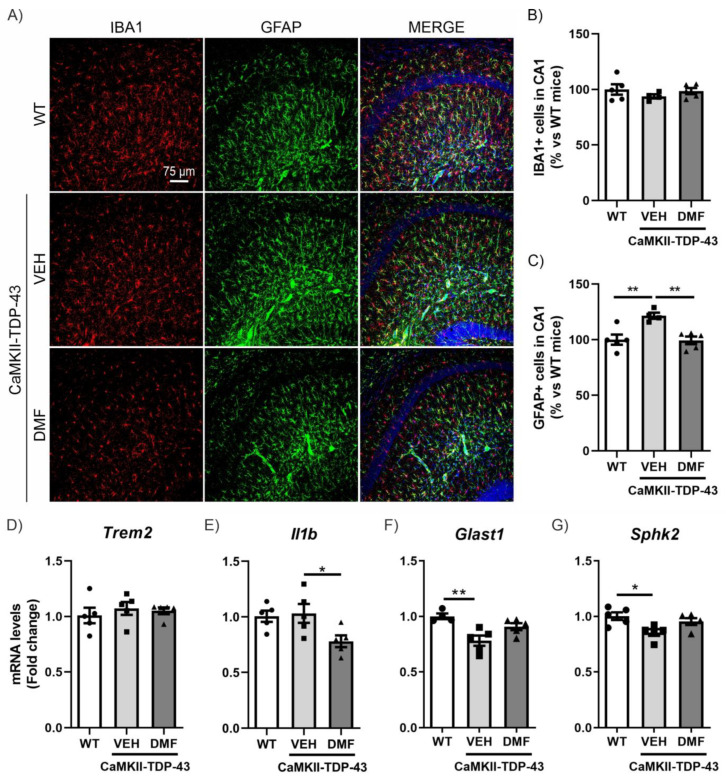
DMF treatment modulates the astrogliosis observed in CAMKII-TDP-43 mice at the hippocampus. (**A**) Immunofluorescence of IBA1 (red) and GFAP (green), microglial and astrocytic markers, respectively, of 30 μm-thick sections in the CA1-hippocampus of mice treated with VEH or DMF, n = 4–5 samples ± SEM. Quantification of number of microglial (**B**) and astrocytes (**C**) cells at the CA1 area of mice treated with VEH or DMF, n = 4–5 samples ± SEM. RT-qPCR determination of mRNA levels of *Trem2* (**D**), *Il1b* (**E**), *Glast1* (**F**), and *Sphk2* (**G**) genes at the hippocampus of mice treated with VEH or DMF, n = 4–5 samples ± SEM. The asterisks represent the difference in significance: * *p* < 0.05, ** *p* < 0.01, comparing each group according to a one-way ANOVA followed by Tukey’s post-test.

To specifically analyze neuroinflammatory processes, we conducted assessments of microgliosis and astrogliosis using immunofluorescence (IF). Microgliosis was analyzed using the IBA1 marker (ionized calcium-binding adapter molecule 1). As shown in Figure 3A,B, we did not observe TDP-43 protein overexpression to result in differences in IBA1-positive cells in the CA1 region of the hippocampus and saw no effects from the DMF treatment. These results were corroborated with the analysis of the mRNA expression levels of *Trem2* and *Il1b* (Figure 3D,E), two markers of microglial function. Triggering receptor expressed on myeloid cells 2 (*Trem2*) is a myeloid cell-specific gene found in brain microglia. It is essential for microglia-mediated synaptic refinement, regulating microglial function and promoting the clearance of neurotoxic substances and abnormal proteins [24,25]. Our results showed no differences between CaMKII-TDP-43-VEH and WT mice (Figure 3D). Furthermore, we did not observe differences in the expression levels of *Il1b* (Figure 3E), which is widely used as pro-inflammatory marker, indicating that the overexpression of TDP-43 does not induce pro-inflammatory activation of microglia. Indeed, we observed that DMF treatment reduced its expression.

Next, we determined astrogliosis through analyzing Glial Fibrillary Acidic Protein (GFAP)-positive cells selectively in the CA1 region of the hippocampus. We observed a significant increase in CaMKII-TDP-43-VEH mice (Figure 3A–C), particularly in the stratum lacunosum-moleculare zone, which was completely reversed by DMF treatment, aligning with the anti-inflammatory effect of DMF reported previously [10]. This hippocampal layer is important as it serves as a link between the entorhinal cortex (EC) and the CA1 hippocampus, and is involved in memory processes, correlating with our NOR results. Additionally, we analyzed the mRNA expression levels of *Glast1* and *Sphk2* to explore this aspect in more detail. Glutamate aspartate transporter 1 (GLAST1) is highly expressed in membranes of astrocytic processes, preferentially around excitatory synapses [26], and it has been described that its down-regulation is a consequence of glutamate-induced neuronal death or the reduction of synaptic activity [27]. Our results are in line with this idea, since in CaMKII-TDP-43-VEH mice we observed a decrease in *Glast1* mRNA levels compared to WT mice (Figure 3F). Furthermore, treatment with DMF prevents this decrease, corroborating the neuroprotective effect of the compound. Additionally, *Sphk2* expression is highest in the brain and is related to anti-inflammatory processes [28,29,30]. CaMKII-TDP-43-VEH mice showed decreased *Sphk2* mRNA levels compared to WT mice (Figure 3G), and these levels were normalized in CaMKII-TDP-43-DMF animals. These data suggest that DMF treatment reduces astrogliosis in CaMKII-TDP-43 mice. We also determined microgliosis and astrogliosis processes in other hippocampal areas, such as CA2-CA3 and the dentate gyrus (DG). In all cases, we did not observe microgliosis or astrogliosis by IF (Figure S4).

### 3.4. DMF Treatment Prevents Degeneration of Layer V in the PFC in CaMKII-TDP-43 Mice

**Figure 4:**
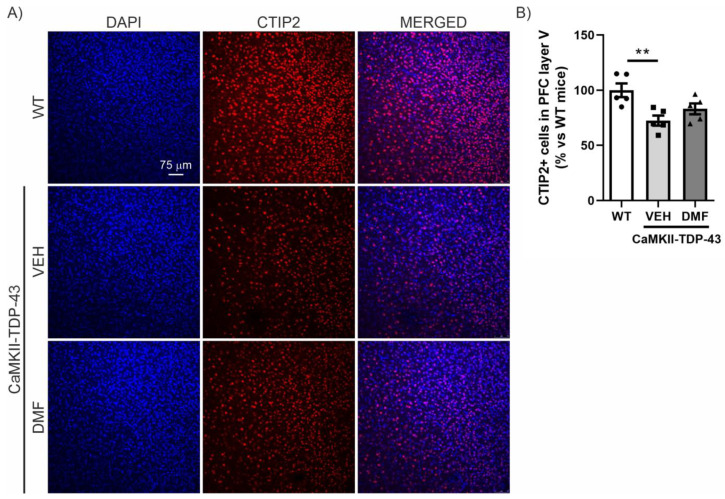
TDP-43 overexpression decreases CTIP2+ neurons and DMF treatment partially reverses this effect in layer V of the cortex. (**A**) Immunofluorescence of CTIP2 (marker of corticospinal motor neurons and other projection neurons in layer V) (red) and DAPI (blue) of 30 μm-thick sections of the mPFC of mice treated with VEH or DMF, n = 5 samples ± SEM. (**B**) Quantification of number of CTIP2+ cells at the layer V of the mPFC of mice treated with VEH or DMF, n= 4–5 samples ± SEM. The asterisks represent the difference in significance: ** *p* < 0.01, comparing each group according to a one-way ANOVA followed by Tukey’s post-test.

We then analyzed the prefrontal cortex (PFC) area, which is the main area associated with neurodegeneration in FTD, where a reduction in CTIP2-positive neurons has previously been described [14,18]. As shown in Figure 4, there is a noticeable reduction in the number of CTIP2-positive neurons in CaMKII-TDP-43-VEH mice compared to WT mice. Treatment with DMF prevents this significant neuronal loss, although the levels do not fully reach those observed in WT mice (Figure 4A,B).

### 3.5. DMF Treatment Avoids Astrogliosis of Layer V in the PFC in CaMKII-TDP-43 Mice

**Figure 5:**
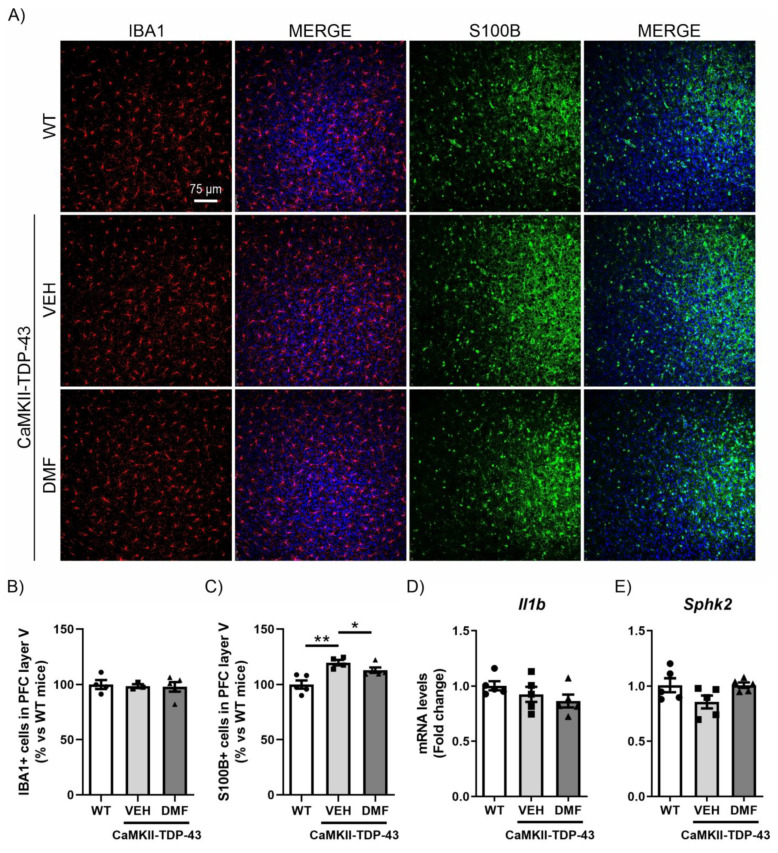
The overexpression of TDP-43 induces astrogliosis in layer V of the mPFC and treatment with DMF partially reverses this effect. (**A**) Immunofluorescence of IBA1 (red) and S100B (green), microglial and astrocytic markers, respectively, of 30 μm-thick sections in the layer V of the mPFC of mice treated with VEH or DMF, n = 4–5 samples ± SEM. Quantification of number of microglial (**B**) and astrocyte (**C**) cells at the layer V of the mPFC of mice treated with VEH or DMF, n = 4–5 samples ± SEM. RT-qPCR determination of mRNA levels of *Il1b* (**D**) and *Sphk2* (**E**) genes in the same area, n = 4–5 samples ± SEM. The asterisks represent the difference in significance: * *p* < 0.05, ** *p* < 0.01, comparing each group according to a one-way ANOVA followed by Tukey’s post-test.

Similar to the study conducted in the hippocampus, we analyzed the inflammatory process in the PFC. We observed that TDP-43 protein overexpression did not produce differences in the number of IBA1-positive cells in layer V of the PFC (Figure 5A,B), and no DMF effects were noticed. For astrogliosis analysis in the PFC, we used the S100-B marker (S100 calcium-binding protein B), a marker of astrocytes particularly recommended for this structure [31]. This choice is due to the different morphological characteristics of astrocytes depending on their brain region, with S100-B immunoreactivity being more intense in the PFC while GFAP immunoreactivity is more pronounced in the hippocampus [14]. IF analysis of S100-B-positive cells, specifically in layer V of the PFC, showed a significant increase in CaMKII-TDP-43-VEH mice (Figure 5A–C), which was partially reversed by DMF treatment. Here, we also analyzed the mRNA expression levels of the pro-inflammatory marker *Il1b* and the anti-inflammatory marker *Sphk2*, finding that the effects were subtler than those observed in the hippocampus (Figure 5D,E).

## 4. Discussion

Currently, there is no cure or specific treatment for FTD. Medications used to treat or slow down AD do not seem to be effective for individuals with FTD, and some may even exacerbate FTD symptoms. Therefore, this work tackles this challenge by repurposing the drug DMF for TDP-43-dependent FTD, which has been shown to slow neurodegeneration in TAU-dependent FTD. This study presents the first evidence that DMF treatment can prevent cognitive decline, neurodegeneration, and astrogliosis in a TDP-43-dependent FTD model. Although further studies at other molecular levels are necessary, our results suggest that DMF treatment could be a promising candidate for slowing down TDP-43-dependent FTD.

It is important to consider that DMF functions as a prodrug, rapidly converting to monomethyl fumarate (MMF) through hydrolysis upon entering the body [32]. Both DMF and MMF trigger the activation of the NRF2 pathway. The primary chemical characteristic of DMF and MMF is their electrophilic nature, stemming from a double bond between reactive carbons known as Michael acceptors [33,34]. This structure makes DMF and MMF susceptible to nucleophilic attack via Michael addition reactions. Consequently, these compounds can form covalent bonds with cysteine thiol groups in various proteins and with glutathione (GSH). Despite their similarities, DMF and MMF exhibit some distinct properties. Notable differences exist in their reactivity with GSH and in their S-alkylation mechanisms. Specifically, MMF demonstrates a significantly slower reaction rate compared to DMF [33]. Although both compounds have similar antioxidant capacities, the main difference lies in the modulation of the pro-inflammatory pathway regulated by the transcription factor NF-κB. In this case, DMF has been described as capable of exerting anti-inflammatory effects through its covalent modulation of p65 [35], while MMF cannot reproduce this effect [36]. These different modes of action are important when establishing the signaling pathways involved in the beneficial effects of DMF/MMF in our TDP-43 FTD model, which we will discuss further below. Previous studies by our group demonstrated a significant decrease only in *Nqo1* mRNA expression levels measured by qPCR and confirmed by RNAscope in this TDP-43 FTD model [11]. This mRNA reduction also reflected a protein level decrease of approximately 50%. These findings indicated that TDP-43 overexpression in the transgenic mice did not induce significant changes in NRF2 antioxidant signaling, although it did significantly decrease NQO1 levels. Recently, NQO1 has been described as an RNA-binding protein involved in translation control [37]. Given that TDP-43 regulates RNA metabolism and trafficking and interacts with several proteins involved in RNA processing, it cannot be ruled out that TDP-43 overexpression specifically modulates NQO1. In this study, we observed a significant decrease in *Nqo1* mRNA levels, and DMF treatment not only normalized them but even elevated them above those of WT or CaMKII-TDP-43-VEH mice, enhancing the protective effect of this enzyme. Considering that there are hardly any studies analyzing the role of NQO1 in the context of wild-type TDP-43 protein, it will be interesting to delve deeper into this aspect in future experiments.

Our data show that TDP-43 overexpression induces astrogliosis but not microgliosis in the PFC and stratum lacunosum-moleculare. This may be due to the different roles of these cell types. Astrocytes are highly specialized glial cells responsible for the trophic and metabolic support of neurons. They are involved in ionic homeostasis, regulation of cerebral blood flow and metabolism, modulation of synaptic activity through neurotransmitter uptake and recycling, and maintenance of the blood-brain barrier [38,39,40]. In contrast, microglia coordinate the removal of synaptic connections, maintenance of brain homeostasis through the regulation of neuronal function, and clearance of protein aggregates throughout life [41,42,43]. In this mouse model, we observed not only an increase in the number of astrocytes but also changes in their morphology and in the expression of astrocytic glutamate transporters, GLAST-1, indicating that the overexpression of TDP-43 induces dysfunctional astrocytes. It has been described that astrocytes exhibit vulnerability to the TDP-43 protein. On the one hand, the dysregulation of TDP-43 in astrocytes leads to cognitive impairment [44], and on the other hand, the loss of TDP-43 in astrocytes results in changes in status of microglia, astrocytes, and oligodendrocytes [45]. These findings suggest different responses between astrocytes and neurons in relation to the TDP-43 protein [46]. This is very important in relation to NRF2 signaling, as it has been reported that astrocytes exhibit poor mitochondrial respiration along with high ROS production, while neurons display high mitochondrial respiration and low ROS production [47,48]. Consequently, it is plausible that the NRF2 signaling pathway is predominantly involved in astrocytes and microglia, and its disruption is linked to changes in the neuroinflammatory process [9,13,49]. However, there is ongoing debate in the field regarding whether NRF2 activation is exclusive to glial cells or if neurons may also participate [50]. Furthermore, our data indicate that DMF treatment significantly reduces astrogliosis in the PFC and stratum lacunosum-moleculare, possibly by enhancing the expression of anti-inflammatory genes such as SPHK2, providing further evidence of the neuroprotective effect of this compound against TDP-43-dependent FTD. Regarding microgliosis, we did not observe any changes associated with the overexpression of TDP-43. It is important to mention that these results have been obtained in males, and the same experiment needs to be conducted in females to determine the effectiveness of DMF treatment regardless of sex in order to extrapolate it to patients.

Regarding oxidative stress/ROS and its implication in TDP-43-dependent FTD, there are no studies identifying a direct relationship, although it is speculated that there may be an increase, similar to what is observed in TAU-dependent FTD [9,10,51]. Therefore, it would be valuable to conduct further studies that delve into this aspect.

Although the NRF2 pathway is one of the main mechanisms of DMF action, this compound also has NRF2-independent effects. For instance, DMF has been described as an inhibitor of GASDERMIN D [52,53], which is involved in the pyroptosis process associated with microglia [54]. The modulation of GASDERMIN D may mediate some of the anti-inflammatory effects of DMF. Unpublished results from our group indicate that pyroptosis is not induced in this TDP-43 overexpression model, suggesting that the anti-inflammatory effect we observe is not due to the inhibition of this pathway.

**Figure 6:**
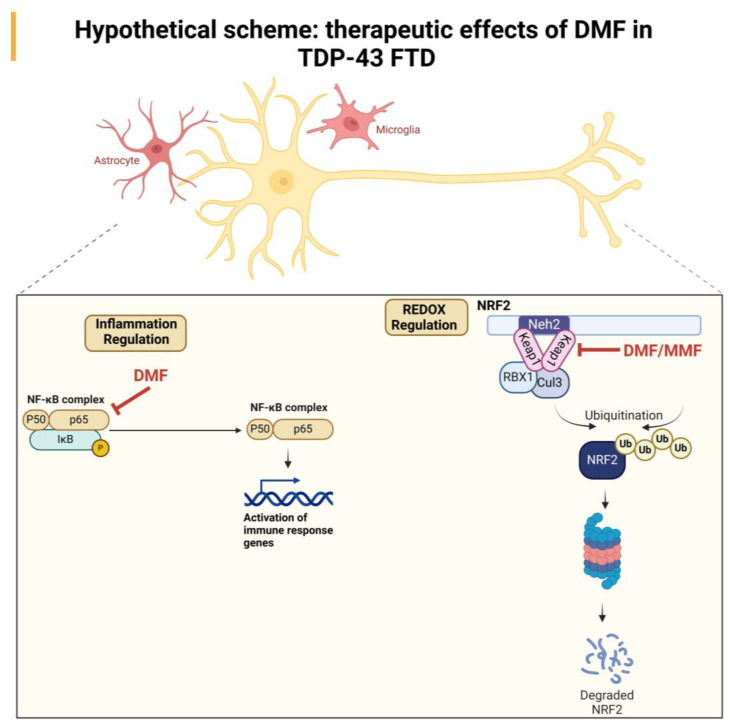
Hypothetical scheme of the neuroprotective effect of DMF treatment in a TDP-43-dependent FTD model. Our results suggest an NRF2-dependent effect mediated by the actions of DMF and MMF at the antioxidant level and an anti-inflammatory effect, which may be primarily mediated by DMF.

All these data indicate that the neuroprotective effect of DMF treatment may be due to a combination of antioxidant and anti-inflammatory effects mediated by the direct action of DMF or its metabolite MMF. A hypothetical scheme that shows the ROS/antioxidant/anti-inflammatory effect in the therapeutic effect of DMF is included in Figure 6.

Another aspect that must also be considered is the possibility that DMF treatment may modulate the expression or localization of the TDP-43 protein. Preliminary studies (which we have not yet published) suggest that DMF treatment does not influence the phosphorylation levels of TDP-43, but we cannot rule out that the treatment may modulate the expression or localization of TDP-43 or other proteins upstream or downstream of the signaling pathways in which TDP-43 is involved. This point could be of vital importance for the clinical relevance of DMF treatment for FTD. This will need to be analyzed in future experiments.

However, DMF treatment has several side-effects in humans. It has been reported that the capsule formulation caused adverse reactions such as flushing and gastrointestinal (GI) issues. The GI events included symptoms such as abdominal pain (both general and upper), diarrhea, and nausea. For the tablet formulation, the most frequently reported adverse reactions were GI events, followed by flushing and lymphopenia [55]. Therefore, other DMF derivatives, such as diroximel fumarate (Vumerity), and other NRF2 activators, such as omaveloxolone, which are currently being used in clinical practice, are now being analyzed [56,57,58].

## 5. Conclusions

This research indicates that DMF therapy mitigates cognitive decline, neurodegeneration, and excessive astrocyte activation in the PFC and stratum lacunosum-moleculare caused by TDP-43 overexpression in a mouse model of FTD. However, further studies are needed to fully elucidate the mechanisms underlying these beneficial effects.

## Figures and Tables

**Table 1:** List of primers used in this study.

Gene Product	Forward Primer	Reverse Primer
*Actb*	5′ TCCTTCCTGGGCATGGAG 3′	5′ AGGAGGAGCAATGATCTTGATCTT 3′
*Gapdh*	5′ CGACTTCAACAGCAACTCCCACTCTTCC 3′	5′ TGGGTGGTCCAGGGTTTCTTACTCCTT 3′
*Glast1*	5′ AATTCACCTCCACGTAGCCC 3′	5′ TCCTTGCGTGTCAGTGTCTT 3′
*Gpx1*	5′ GGACTACACCGAGATGAACG 3′	5′ GATGTACTTGGGGTCGGTCA 3′
*Hmox1*	5′ CACAGATGGCGTCACTTCGTC 3′	5′ GTGAGGACCCACTGGAGGAG 3′
*Il1b*	5′ CTGGTGTGTGACGTTCCCATTA 3′	5′ CCGACAGCACGAGGCTTT 3′
*Nfe2l2*	5′ CCCGAAGCACCCTGAAGGCA 3′	5′ CCAGGCGGTGGGTCTCCGTA 3′
*Nqo1*	5′ GGTAGCGGCTCCATGTACTC 3′	5′ CATCCTTCCAGGATCTGCAT 3′
*Sphk2*	5′ ATATTCATGGGGGCGTGTCCCAG 3′	5′ TCCTGGACCAGCCTCTGGGTGT 3′
*Tbp*	5′ TGCACAGGAGCCAAGAGTGAA 3′	5′ CACATCACAGCTCCCCACCA 3′
*Trem2*	5′ TGCAGAAAGTACTGGTGGAGGT 3′	5′ CTAGAGGTGACCCACAGGATGAAA 3′
*Txn1*	5′ CTCCCCGCAACAGCCAAAAT 3′	5′ CAGAGAAGTCCACCACGACAA 3′

**Table 2:** List of antibodies used in this study.

Antibody	Source	Catalog Number	Dilution
Alexa Fluor 488 donkey anti-mouse IgG	Thermo Fisher Scientific, Waltham, MA, USA	A21202	1:500 IF
Alexa Fluor 488 donkey anti-rabbit IgG	Thermo Fisher Scientific	A21206	1:500 IF
Alexa Fluor 546 donkey anti-mouse IgG	Thermo Fisher Scientific	A31570	1:500 IF
Alexa Fluor 546 donkey anti-rabbit IgG	Thermo Fisher Scientific	A31572	1:500 IF
CALBINDIN-D28K	Synaptic Systems, Göttingen, Germany	214.002	1:500 IF
CTIP2	Abcam, Cambridge, UK	ab28448	1:400 IF
GFAP	Sigma-Aldrich, St. Louis, MO, USA	G3893	1:400 IF
IBA1	Wako Chemicals, Richmond, VA, USA	019-19741	1:500 IF
S100B	Abcam	ab52642	1:500 IF

## Data Availability

Data are contained within the article and Supplementary Materials.

## Supplementary Materials

The following supporting information can be downloaded at: https://www.mdpi.com/article/10.3390/antiox13091072/s1, Figure S1: Induction of the NRF2 pathway by DMF in the hippocampus; Figure S2: CALBINDIN-D28K levels in the CA1 and CA2 regions of the hippocampus; Figure S3: CALBINDIN-D28K levels in the CA3 region of the hippocampus; Figure S4: Analysis of microgliosis and astrogliosis in the CA2-CA3 and dentate gyrus of the hippocampus.

## References

[1] Leroy M., Bertoux M., Skrobala E., Mode E., Adnet-Bonte C., Le Ber I., Bombois S., Cassagnaud P., Chen Y., Deramecourt V. (2021). Characteristics and progression of patients with frontotemporal dementia in a regional memory clinic network. Alzheimer’s Res. Ther..

[2] Grossman M., Seeley W.W., Boxer A.L., Hillis A.E., Knopman D.S., Ljubenov P.A., Miller B., Piguet O., Rademakers R., Whitwell J.L. (2023). Frontotemporal lobar degeneration. Nat. Rev. Dis. Primers.

[3] Tong K.I., Padmanabhan B., Kobayashi A., Shang C., Hirotsu Y., Yokoyama S., Yamamoto M. (2007). Different electrostatic potentials define ETGE and DLG motifs as hinge and latch in oxidative stress response. Mol. Cell. Biol..

[4] Itoh K., Chiba T., Takahashi S., Ishii T., Igarashi K., Katoh Y., Oyake T., Hayashi N., Satoh K., Hatayama I. (1997). An Nrf2/small Maf heterodimer mediates the induction of phase II detoxifying enzyme genes through antioxidant response elements. Biochem. Biophys. Res. Commun..

[5] Itoh K., Wakabayashi N., Katoh Y., Ishii T., Igarashi K., Engel J.D., Yamamoto M. (1999). Keap1 represses nuclear activation of antioxidant responsive elements by Nrf2 through binding to the amino-terminal Neh2 domain. Genes. Dev..

[6] Hayes J.D., Dinkova-Kostova A.T. (2014). The Nrf2 regulatory network provides an interface between redox and intermediary metabolism. Trends Biochem. Sci..

[7] Cuadrado A. (2015). Structural and functional characterization of Nrf2 degradation by glycogen synthase kinase 3/β-TrCP. Free Radic. Biol. Med..

[8] Hayes J.D., Chowdhry S., Dinkova-Kostova A.T., Sutherland C. (2015). Dual regulation of transcription factor Nrf2 by Keap1 and by the combined actions of β-TrCP and GSK-3. Biochem. Soc. Trans..

[9] Lastres-Becker I., Innamorato N.G., Jaworski T., Rabano A., Kugler S., Van Leuven F., Cuadrado A. (2014). Fractalkine activates NRF2/NFE2L2 and heme oxygenase 1 to restrain tauopathy-induced microgliosis. Brain.

[10] Cuadrado A., Kugler S., Lastres-Becker I. (2018). Pharmacological targeting of GSK-3 and NRF2 provides neuroprotection in a preclinical model of tauopathy. Redox Biol..

[11] Lastres-Becker I., de Lago E., Martínez A., Fernández-Ruiz J. (2022). New Statement about NRF2 in Amyotrophic Lateral Sclerosis and Frontotemporal Dementia. Biomolecules.

[12] Tsai K.J., Yang C.H., Fang Y.H., Cho K.H., Chien W.L., Wang W.T., Wu T.W., Lin C.P., Fu W.M., Shen C.K. (2010). Elevated expression of TDP-43 in the forebrain of mice is sufficient to cause neurological and pathological phenotypes mimicking FTLD-U. J. Exp. Med..

[13] Lastres-Becker I., Garcia-Yague A.J., Scannevin R.H., Casarejos M.J., Kugler S., Rabano A., Cuadrado A. (2016). Repurposing the NRF2 Activator Dimethyl Fumarate as Therapy Against Synucleinopathy in Parkinson’s Disease. Antioxid. Redox Signal..

[14] Santos-García I., Rodríguez-Cueto C., Villegas P., Piscitelli F., Lauritano A., Shen C.J., Di Marzo V., Fernández-Ruiz J., de Lago E. (2023). Preclinical investigation in FAAH inhibition as a neuroprotective therapy for frontotemporal dementia using TDP-43 transgenic male mice. J. Neuroinflamm..

[15] Curtis M.J., Alexander S., Cirino G., Docherty J.R., George C.H., Giembycz M.A., Hoyer D., Insel P.A., Izzo A.A., Ji Y. (2018). Experimental design and analysis and their reporting II: Updated and simplified guidance for authors and peer reviewers. Br. J. Pharmacol..

[16] Galán-Ganga M., Rodríguez-Cueto C., Merchán-Rubira J., Hernández F., Ávila J., Posada-Ayala M., Lanciego J.L., Luengo E., Lopez M.G., Rábano A. (2021). Cannabinoid receptor CB2 ablation protects against TAU induced neurodegeneration. Acta Neuropathol. Commun..

[17] Gundersen H.J., Jensen E.B. (1987). The efficiency of systematic sampling in stereology and its prediction. J. Microsc..

[18] Gonzalo-Consuegra C., Santos-García I., García-Toscano L., Martín-Baquero R., Rodríguez-Cueto C., Wittwer M.B., Dzygiel P., Grether U., de Lago E., Fernández-Ruiz J. (2024). Involvement of CB(1) and CB(2) receptors in neuroprotective effects of cannabinoids in experimental TDP-43 related frontotemporal dementia using male mice. Biomed. Pharmacother..

[19] Deng W., Aimone J.B., Gage F.H. (2010). New neurons and new memories: How does adult hippocampal neurogenesis affect learning and memory?. Nat. Rev. Neurosci..

[20] Li J.T., Xie X.M., Yu J.Y., Sun Y.X., Liao X.M., Wang X.X., Su Y.A., Liu Y.J., Schmidt M.V., Wang X.D. (2017). Suppressed Calbindin Levels in Hippocampal Excitatory Neurons Mediate Stress-Induced Memory Loss. Cell Rep..

[21] Palop J.J., Jones B., Kekonius L., Chin J., Yu G.Q., Raber J., Masliah E., Mucke L. (2003). Neuronal depletion of calcium-dependent proteins in the dentate gyrus is tightly linked to Alzheimer’s disease-related cognitive deficits. Proc. Natl. Acad. Sci. USA.

[22] Bright F., Werry E.L., Dobson-Stone C., Piguet O., Ittner L.M., Halliday G.M., Hodges J.R., Kiernan M.C., Loy C.T., Kassiou M. (2019). Neuroinflammation in frontotemporal dementia. Nat. Rev. Neurol..

[23] Lemprière S. (2023). Neuroinflammation predicts cognitive decline in FTD. Nat. Rev. Neurol..

[24] Tagliatti E., Desiato G., Mancinelli S., Bizzotto M., Gagliani M.C., Faggiani E., Hernández-Soto R., Cugurra A., Poliseno P., Miotto M. (2024). Trem2 expression in microglia is required to maintain normal neuronal bioenergetics during development. Immunity.

[25] Li Y., Xu H., Wang H., Yang K., Luan J., Wang S. (2023). TREM2: Potential therapeutic targeting of microglia for Alzheimer’s disease. Biomed. Pharmacother..

[26] Achicallende S., Bonilla-Del Río I., Serrano M., Mimenza A., Lekunberri L., Anaut-Lusar I., Puente N., Gerrikagoitia I., Grandes P. (2022). GLAST versus GFAP as astroglial marker for the subcellular study of cannabinoid CB(1) receptors in astrocytes. Histochem. Cell Biol..

[27] Perego C., Vanoni C., Bossi M., Massari S., Basudev H., Longhi R., Pietrini G. (2000). The GLT-1 and GLAST glutamate transporters are expressed on morphologically distinct astrocytes and regulated by neuronal activity in primary hippocampal cocultures. J. Neurochem..

[28] Weigert A., von Knethen A., Thomas D., Faria I., Namgaladze D., Zezina E., Fuhrmann D., Petcherski A., Heringdorf D.M.Z., Radeke H.H. (2019). Sphingosine kinase 2 is a negative regulator of inflammatory macrophage activation. Biochim. Biophys. Acta Mol. Cell Biol. Lipids.

[29] Standoli S., Rapino C., Di Meo C., Rudowski A., Kämpfer-Kolb N., Volk L.M., Thomas D., Trautmann S., Schreiber Y., Meyer Zu Heringdorf D. (2023). Sphingosine Kinases at the Intersection of Pro-Inflammatory LPS and Anti-Inflammatory Endocannabinoid Signaling in BV2 Mouse Microglia Cells. Int. J. Mol. Sci..

[30] Liu H., Sugiura M., Nava V.E., Edsall L.C., Kono K., Poulton S., Milstien S., Kohama T., Spiegel S. (2000). Molecular cloning and functional characterization of a novel mammalian sphingosine kinase type 2 isoform. J. Biol. Chem..

[31] Aguilera-Portillo G., Rangel-López E., Villeda-Hernández J., Chavarría A., Castellanos P., Elmazoglu Z., Karasu Ç., Túnez I., Pedraza G., Königsberg M. (2019). The Pharmacological Inhibition of Fatty Acid Amide Hydrolase Prevents Excitotoxic Damage in the Rat Striatum: Possible Involvement of CB1 Receptors Regulation. Mol. Neurobiol..

[32] Majkutewicz I. (2022). Dimethyl fumarate: A review of preclinical efficacy in models of neurodegenerative diseases. Eur. J. Pharmacol..

[33] Schmidt T.J., Ak M., Mrowietz U. (2007). Reactivity of dimethyl fumarate and methylhydrogen fumarate towards glutathione and N-acetyl-l-cysteine—Preparation of S-substituted thiosuccinic acid esters. Bioorganic Med. Chem..

[34] Piroli G.G., Manuel A.M., Patel T., Walla M.D., Shi L., Lanci S.A., Wang J., Galloway A., Ortinski P.I., Smith D.S. (2019). Identification of Novel Protein Targets of Dimethyl Fumarate Modification in Neurons and Astrocytes Reveals Actions Independent of Nrf2 Stabilization*[S]. Mol. Cell. Proteom..

[35] Kastrati I., Siklos M.I., Calderon-Gierszal E.L., El-Shennawy L., Georgieva G., Thayer E.N., Thatcher G.R.J., Frasor J. (2016). Dimethyl Fumarate Inhibits the Nuclear Factor κB Pathway in Breast Cancer Cells by Covalent Modification of p65 Protein*. J. Biol. Chem..

[36] Gillard G.O., Collette B., Anderson J., Chao J., Scannevin R.H., Huss D.J., Fontenot J.D. (2015). DMF, but not other fumarates, inhibits NF-κB activity in vitro in an Nrf2-independent manner. J. Neuroimmunol..

[37] Di Francesco A., Di Germanio C., Panda A.C., Huynh P., Peaden R., Navas-Enamorado I., Bastian P., Lehrmann E., Diaz-Ruiz A., Ross D. (2016). Novel RNA-binding activity of NQO1 promotes SERPINA1 mRNA translation. Free Radic. Biol. Med..

[38] Allen N.J., Barres B.A. (2009). Glia—More than just brain glue. Nature.

[39] Lee H.-G., Wheeler M.A., Quintana F.J. (2022). Function and therapeutic value of astrocytes in neurological diseases. Nat. Rev. Drug Discov..

[40] Gradisnik L., Velnar T. (2023). Astrocytes in the central nervous system and their functions in health and disease: A review. World J. Clin. Cases.

[41] Colonna M., Butovsky O. (2017). Microglia Function in the Central Nervous System During Health and Neurodegeneration. Annu. Rev. Immunol..

[42] Lenz K.M., Nelson L.H. (2018). Microglia and Beyond: Innate Immune Cells As Regulators of Brain Development and Behavioral Function. Front. Immunol..

[43] Augusto-Oliveira M., Arrifano G.P., Lopes-Araújo A., Santos-Sacramento L., Takeda P.Y., Anthony D.C., Malva J.O., Crespo-Lopez M.E. (2019). What Do Microglia Really Do in Healthy Adult Brain?. Cells.

[44] Licht-Murava A., Meadows S.M., Palaguachi F., Song S.C., Jackvony S., Bram Y., Zhou C., Schwartz R.E., Froemke R.C., Orr A.L. (2023). Astrocytic TDP-43 dysregulation impairs memory by modulating antiviral pathways and interferon-inducible chemokines. Sci. Adv..

[45] Peng A.Y.T., Agrawal I., Ho W.Y., Yen Y.C., Pinter A.J., Liu J., Phua Q.X.C., Koh K.B., Chang J.C., Sanford E. (2020). Loss of TDP-43 in astrocytes leads to motor deficits by triggering A1-like reactive phenotype and triglial dysfunction. Proc. Natl. Acad. Sci. USA.

[46] Smethurst P., Risse E., Tyzack G.E., Mitchell J.S., Taha D.M., Chen Y.R., Newcombe J., Collinge J., Sidle K., Patani R. (2020). Distinct responses of neurons and astrocytes to TDP-43 proteinopathy in amyotrophic lateral sclerosis. Brain.

[47] Lopez-Fabuel I., Le Douce J., Logan A., James A.M., Bonvento G., Murphy M.P., Almeida A., Bolaños J.P. (2016). Complex I assembly into supercomplexes determines differential mitochondrial ROS production in neurons and astrocytes. Proc. Natl. Acad. Sci. USA.

[48] Vicente-Gutierrez C., Bonora N., Jimenez-Blasco D., Lopez-Fabuel I., Bates G., Murphy M.P., Almeida A., Bolaños J.P. (2021). Abrogating mitochondrial ROS in neurons or astrocytes reveals cell-specific impact on mouse behaviour. Redox Biol..

[49] Lastres-Becker I., Ulusoy A., Innamorato N.G., Sahin G., Rábano A., Kirik D., Cuadrado A. (2012). α-Synuclein expression and Nrf2 deficiency cooperate to aggravate protein aggregation, neuronal death and inflammation in early-stage Parkinson’s disease. Hum. Mol. Genet..

[50] Liddell J.R. (2017). Are Astrocytes the Predominant Cell Type for Activation of Nrf2 in Aging and Neurodegeneration?. Antioxidants.

[51] Esteras N., Kopach O., Maiolino M., Lariccia V., Amoroso S., Qamar S., Wray S., Rusakov D.A., Jaganjac M., Abramov A.Y. (2022). Mitochondrial ROS control neuronal excitability and cell fate in frontotemporal dementia. Alzheimers Dement..

[52] Bandharam N., Lockey R.F., Kolliputi N. (2023). Pyroptosis Inhibition in Disease Treatment: Opportunities and Challenges. Cell Biochem. Biophys..

[53] Zhou B., Abbott D.W. (2023). Chemical modulation of gasdermin D activity: Therapeutic implications and consequences. Semin. Immunol..

[54] Wu X., Wan T., Gao X., Fu M., Duan Y., Shen X., Guo W. (2022). Microglia Pyroptosis: A Candidate Target for Neurological Diseases Treatment. Front. Neurosci..

[55] Dimethyl Fumarate (2006). Drugs and Lactation Database (LactMed^®^).

[56] Amoroso R., Maccallini C., Bellezza I. (2023). Activators of Nrf2 to Counteract Neurodegenerative Diseases. Antioxidants.

[57] Gunther K., Lynch D.R. (2024). Pharmacotherapeutic strategies for Friedreich Ataxia: A review of the available data. Expert. Opin. Pharmacother..

[58] Hynes S.M., Goldsberry A., Henneghan P.D., Murai M., Shinde A., Wells J.A., Wu L., Wu T., Zahir H., Khan S. (2024). Relative Bioavailability of Omaveloxolone When Capsules Are Sprinkled Over and Mixed in Applesauce Compared With Administration as Intact Omaveloxolone Capsules: A Phase 1, Randomized, Open-Label, Single-Dose, Crossover Study in Healthy Adults. J. Clin. Pharmacol..

