# Supplementary material for: Beneficial Effect of Dimethyl Fumarate Drug Repositioning in a Mouse Model of TDP-43-Dependent Frontotemporal Dementia
Source: Antioxidants (Basel). 2024 Sep 2;13(9):1072. doi: 10.3390/antiox13091072 (PMC11428793; doi:10.3390/antiox13091072)
Supplement: Supplementary file 1 [file antioxidants-13-01072-s001.zip › antioxidants-3158664-supplementary.pdf]

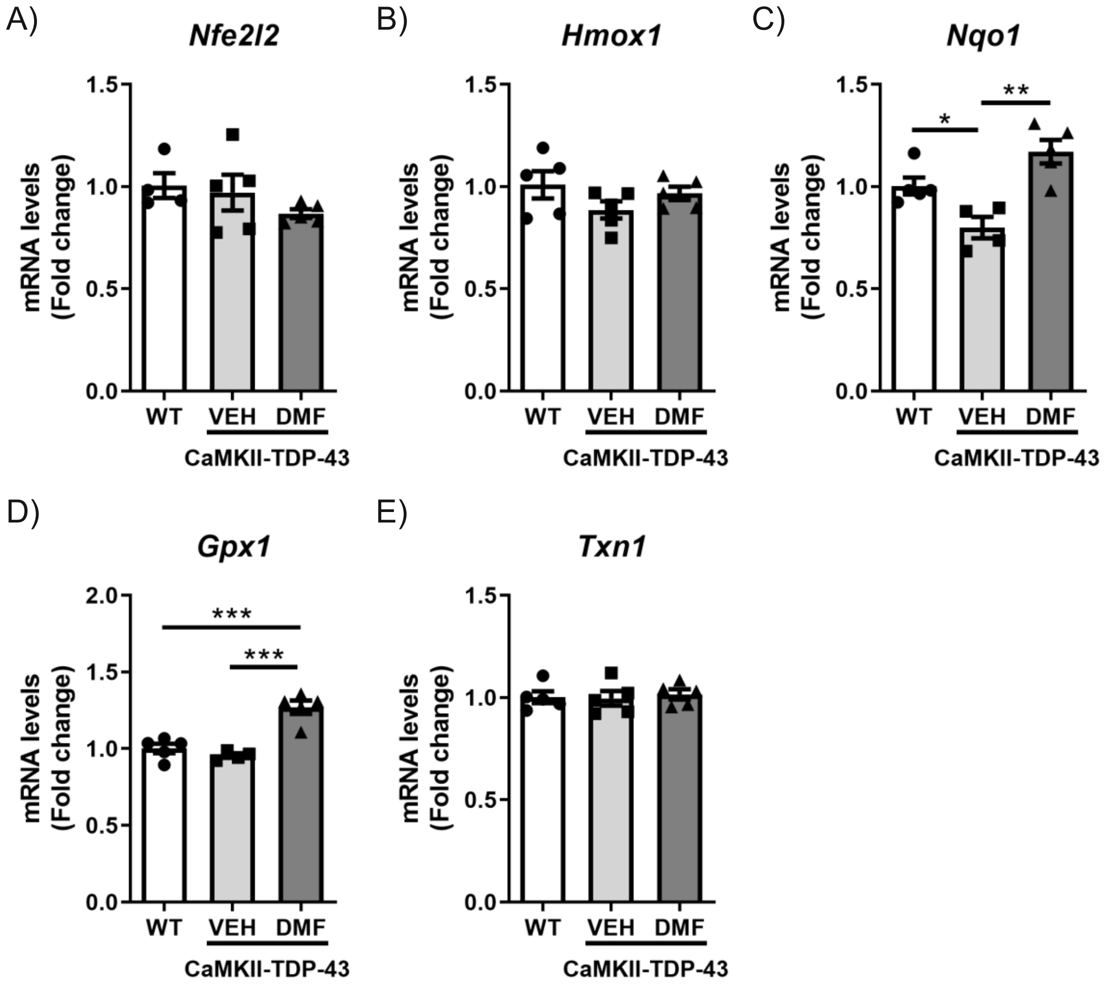
A)
Nfe2l2
B)
Hmox1
C)
Nqo1
D)
Gpx1
E)
Txn1
mRNA levels
(Fold change)
WT
VEH
DMF
CaMKII-TDP-43
*
**
***
***

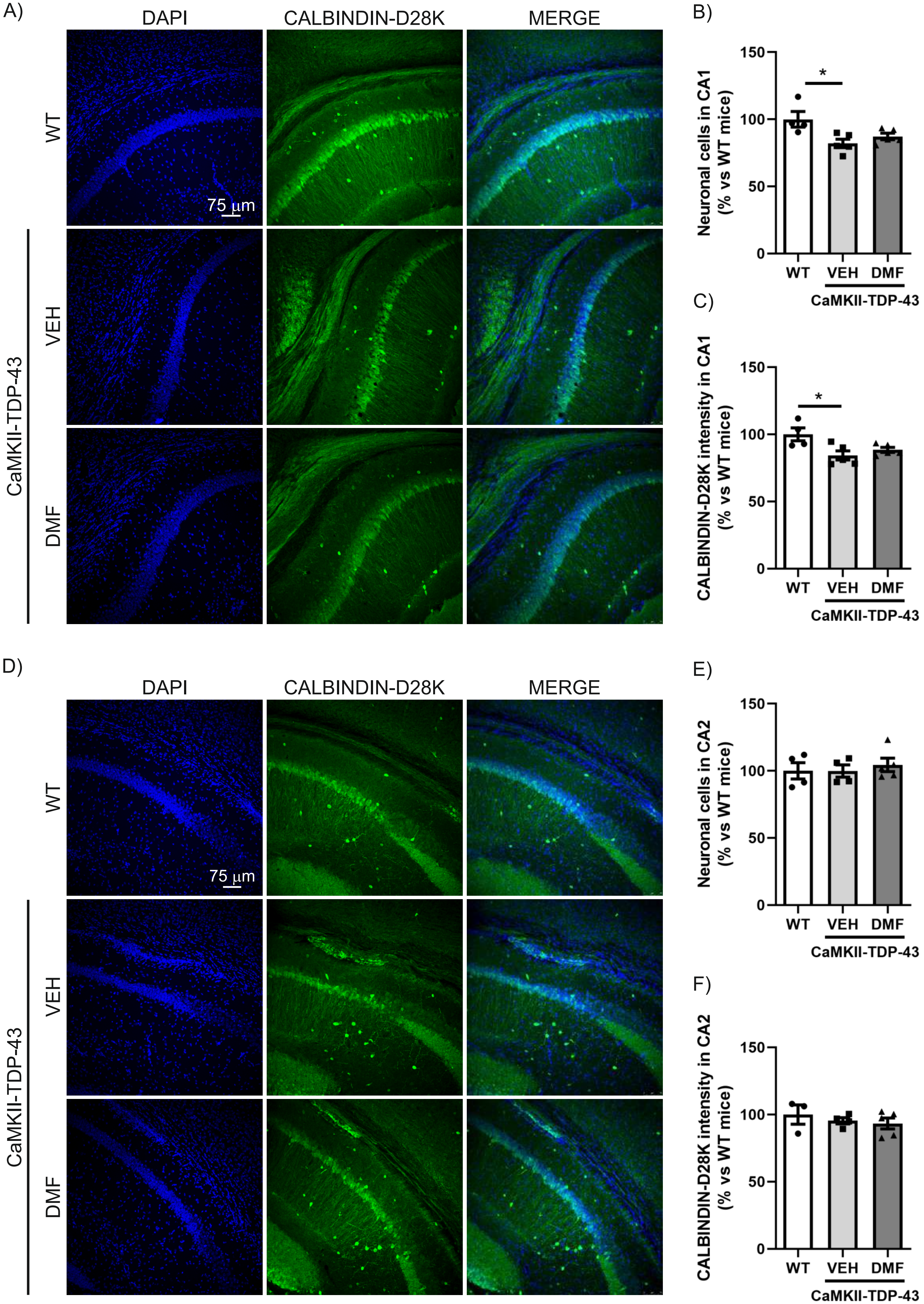

A)
DAPI
CALBINDIN-D28K
MERGE
WT
75 µm
CaMKII-TDP-43
VEH
DMF
B)
Neuronal cells in CA1 (% vs WT mice)
150
100
50
0
*
WT
VEH
DMF
CaMKII-TDP-43
C)
CALBINDIN-D28K intensity in CA1 (% vs WT mice)
150
100
50
0
*
WT
VEH
DMF
CaMKII-TDP-43
D)
DAPI
CALBINDIN-D28K
MERGE
WT
75 µm
CaMKII-TDP-43
VEH
DMF
E)
Neuronal cells in CA2 (% vs WT mice)
150
100
50
0
WT
VEH
DMF
CaMKII-TDP-43
F)
CALBINDIN-D28K intensity in CA2 (% vs WT mice)
150
100
50
0
WT
VEH
DMF
CaMKII-TDP-43

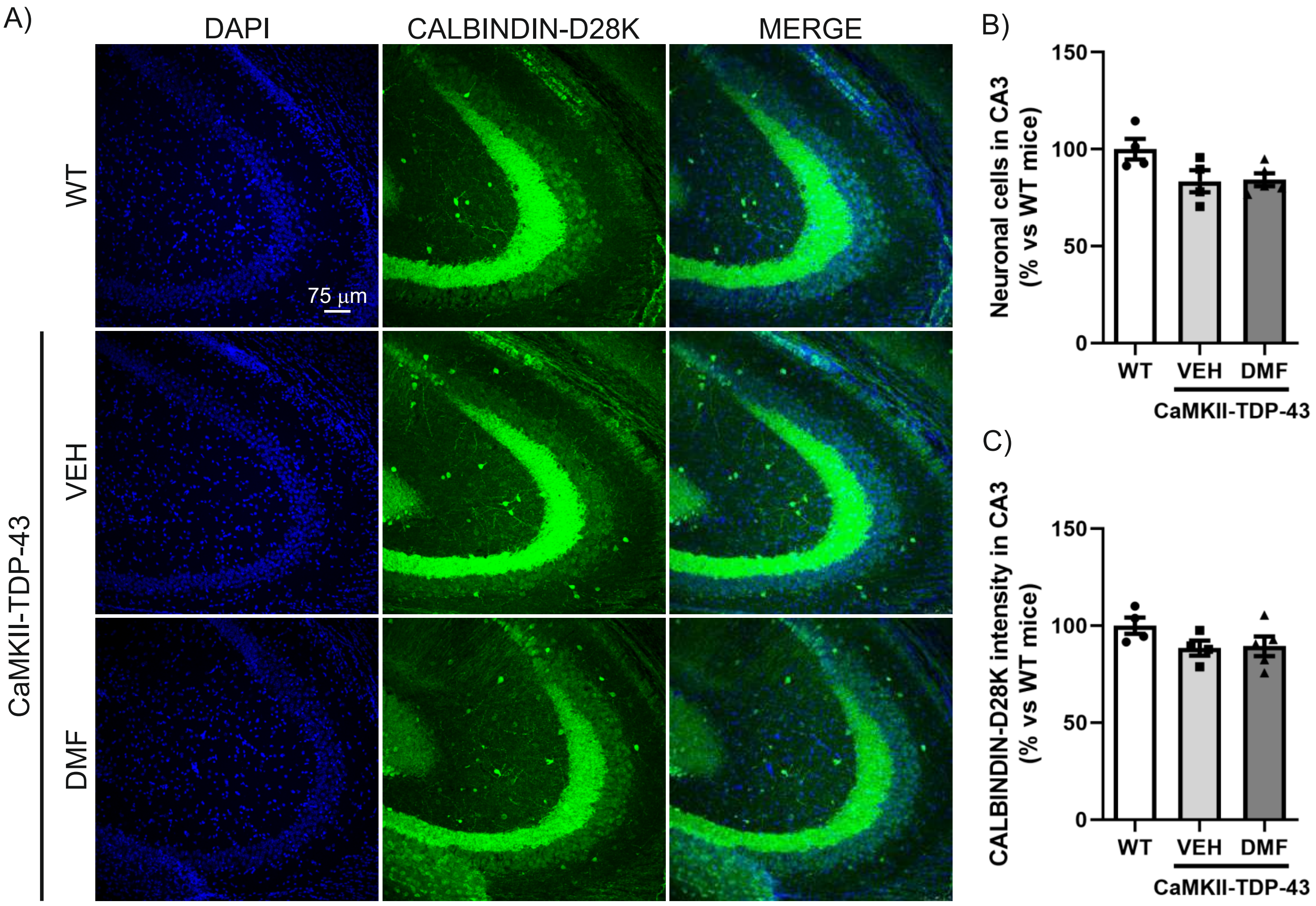

A)
DAPI
CALBINDIN-D28K
MERGE
WT
75 µm
CaMKII-TDP-43
VEH
DMF
B)
Neuronal cells in CA3
(% vs WT mice)
150
100
50
0
WT
VEH
DMF
CaMKII-TDP-43
C)
CALBINDIN-D28K intensity in CA3
(% vs WT mice)
150
100
50
0
WT
VEH
DMF
CaMKII-TDP-43

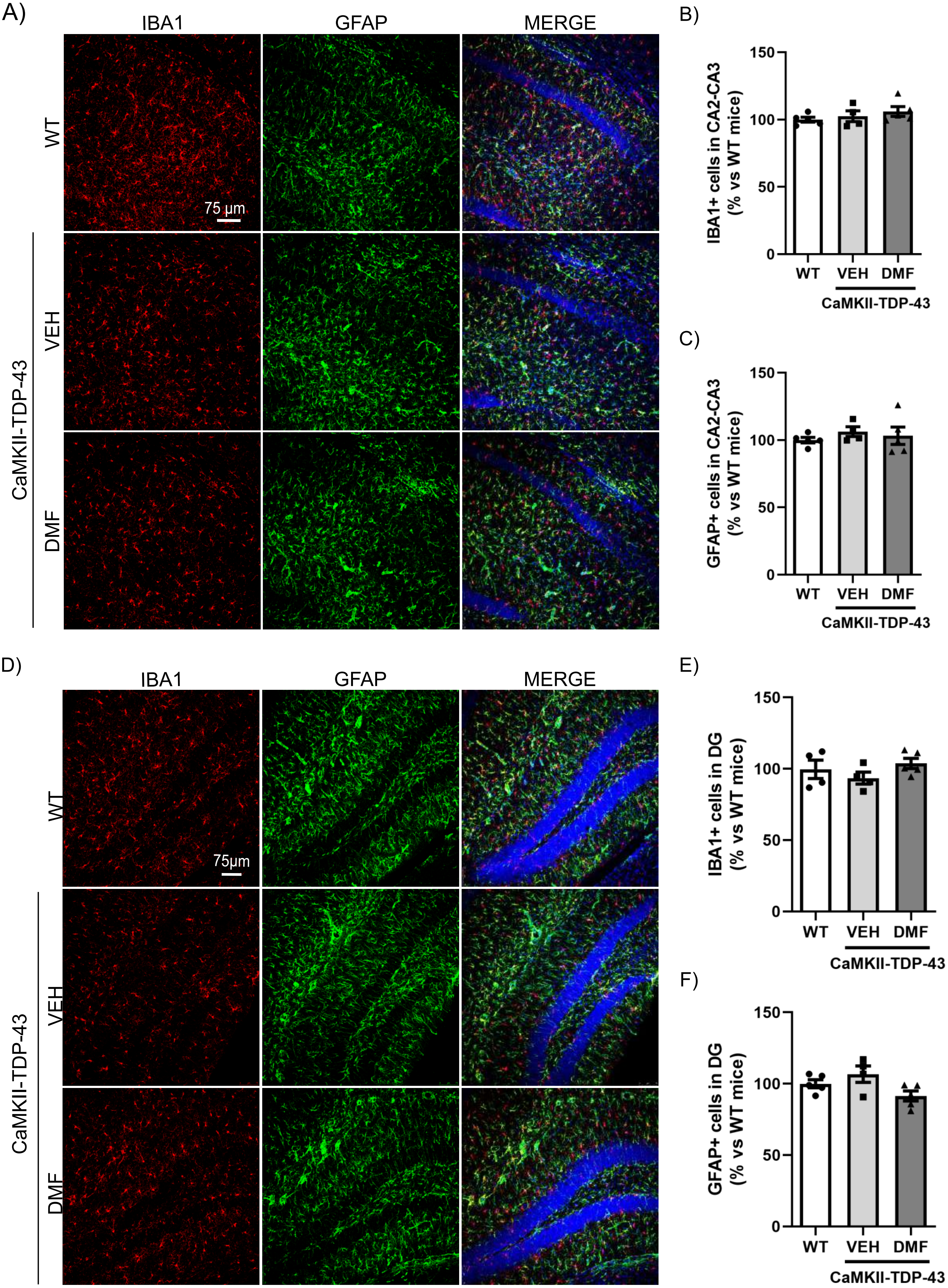

A)
IBA1
GFAP
MERGE
WT
75 µm
CaMKII-TDP-43
VEH
DMF
B)
IBA1+ cells in CA2-CA3 (% vs WT mice)
150
100
50
0
WT
VEH
DMF
CaMKII-TDP-43
C)
GFAP+ cells in CA2-CA3 (% vs WT mice)
150
100
50
0
WT
VEH
DMF
CaMKII-TDP-43
D)
IBA1
GFAP
MERGE
WT
75µm
CaMKII-TDP-43
VEH
DMF
E)
IBA1+ cells in DG (% vs WT mice)
150
100
50
0
WT
VEH
DMF
CaMKII-TDP-43
F)
GFAP+ cells in DG (% vs WT mice)
150
100
50
0
WT
VEH
DMF
CaMKII-TDP-43

Supplemmentary Materials (SF)

Figure S1: The treatment with DMF induces mRNA expression of late-kinetic enzymes of the NRF2 pathway in the hippocampus of CaMKII-TDP-43 mice. RT-qPCR determination of mRNA levels of (**A**) *Nfe2l2*, (**B**) *Hmox1*, (**C**) *Nqo1*, (**D**) *Gpx1* and (**E**) *Txn1* genes at the hippocampus of WT mice and CaMKII-TDP-43 mice treated with VEH or DMF, n = 4–5 samples ± SEM. The asterisks represent the difference in significance: * $p < 0.05$, ** $p < 0.01$, comparing each group according to a one-way ANOVA followed by Tukey’s post-test.

Figure S2: DMF does not have a neuroprotective effect on CA1 and CA2 areas of hippocampus. (**A**) Immunofluorescence staining of DAPI and CALBINDIN-D28K of 30 μm-thick sections of CA1 of the hippocampus from WT, and mice treated with vehicle or DMF. (**B**) Quantification of the neuronal cells stained with DAPI in CA1 from WT, and CaMKII-TDP-43 mice treated with vehicle or DMF. (**C**) Quantification of the intensity of the area stained with CALBINDIN-D28K in CA1 from WT, and CaMKII-TDP-43 mice treated with vehicle or DMF. (**D**) Immunofluorescence staining of DAPI and CALBINDIN-D28K of 30 μm-thick sections of CA2 of the hippocampus from WT, and CaMKII-TDP-43 mice treated with vehicle or DMF. (**E**) Quantification of the neuronal cells stained with DAPI in CA2 from WT, and CaMKII-TDP-43 mice treated with vehicle or DMF. (**F**) Quantification of the intensity of the area stained with CALBINDIN-D28K in CA2 from WT, and CaMKII-TDP-43 mice treated with vehicle or DMF. Bars indicate the mean of n = 4–5 samples ± SEM. Asterisks show significant differences with * $p < 0.05$ comparing each group according to a one-way ANOVA followed by Tukey’s post-test.

Figure S3: The treatment with DMF does not exert a neuroprotective effect on CA3 area of hippocampus. (**A**) Immunofluorescence staining of DAPI and CALBINDIN-D28K of 30 μm-thick sections of CA3 of the hippocampus from WT, and CaMKII-TDP-43 mice treated with vehicle or DMF. (**B**) Quantification of the neuronal cells stained with DAPI in CA3 from WT, and CaMKII-TDP-43 mice treated with vehicle or DMF. (**C**) Quantification of the intensity of the area stained with CALBINDIN-D28K in CA3 from WT, and CaMKII-TDP-43 mice treated with vehicle or DMF. Bars indicate the mean of n = 4–5 samples ± SEM. Asterisks show significant differences with * $p < 0.05$ comparing each group according to a one-way ANOVA followed by Tukey’s post-test.

Figure S4: DMF treatment does not modulate neuroinflammation in CA2, CA3 and dentate gyrus of hippocampus of CaMKII-TDP-43 mice. (**A**) Immunofluorescence of IBA1 (red) and GFAP (green), microglial and astrocytic markers, respectively, of 30 μm-thick sections in the CA2-CA3 regions of hippocampus of WT mice and CaMKII-TDP-43 mice treated with VEH or DMF, n = 4–5 samples ± SEM. (**B**) Quantification of number of $IBA1^+$ microglial cells and (**C**) $GFAP^+$ astrocytes cells at the CA2-CA3 regions of WT mice and CaMKII-TDP-43 mice treated with VEH or DMF, n = 4–5 samples ± SEM. (**D**) Immunofluorescence of IBA1 (red) and GFAP (green) of 30 μm-thick sections in the dentate gyrus area of hippocampus of WT mice and CaMKII-TDP-43 mice treated with VEH or DMF, n = 4–5 samples ± SEM. (**E**) Quantification of number of $IBA1^+$ microglial cells and (**F**) $GFAP^+$ astrocytes cells at the dentate gyrus of WT mice and CaMKII-TDP-43 mice treated with VEH or DMF, n = 4–5 samples ± SEM. The asterisks represent the difference in significance: * $p < 0.05$, ** $p < 0.01$, comparing each group according to a one-way ANOVA followed by Tukey’s post-test.
